# Impact of COVID-19 on Medical Supply in Adults With Congenital Heart Disease

**DOI:** 10.3389/fpsyt.2022.812611

**Published:** 2022-03-17

**Authors:** Steffen Akkermann, Tim Halling, Friederike Löffler, Ann S. Silber-Peest, Tillmann Krüger, Stefan Bleich, Johann Bauersachs, Kai G. Kahl, Mechthild Westhoff-Bleck

**Affiliations:** ^1^Department of Psychiatry, Social Psychiatry and Psychotherapy, Hannover Medical School, Hannover, Germany; ^2^Department of Cardiology and Angiology, Hannover Medical School, Hannover, Germany

**Keywords:** adult congenital heart disease (ACHD), COVID-19, depression and anxiety disorders, adherence, medical care

## Abstract

**Introduction:**

In March 2020, the World Health organization declared COVID-19 a global pandemic. One year later, the direct and indirect burden of the COVID-19 pandemic become more visible. In this context, there is concern about the allocation of medical resources and medical treatment of other diseases than COVID-19. Particularly, patients with chronic diseases need constant medical and pharmacological treatment. Therefore, we evaluated a large cohort of patients with adult congenital heart disease (ACHD) regarding postponed medical appointments and their possibilities to receive medical treatment during the COVID-19 pandemic.

**Methods:**

This cross-sectional study included 559 patients with ACHD (mean age 37.32 ± 11.98; 47% female). Clinical characteristics, answers to questionnaires concerning lifestyle, psychological well-being, addictive behavior and adherence were related to postponed medical appointments and limited access to medical care.

**Results:**

One hundred and nine patients (19.5%) reported problems getting necessary medical treatment or visiting a physician. Higher anxiety levels (*p* = 0.004) emerged as the main factor associated with medical undertreatment. The main risk factors for postponement of least one medical appointment (*n* = 91) were higher depression (*p* = 0.013) and anxiety (*p* = 0.05) symptoms as well as female sex (*p* ≤ 0.0001) and documented arrhythmias (*p* = 0.007) indicating a particular risk group of cardiovascular complications. In contrast, frequent physical activity identified patients at lower risk.

**Conclusion:**

In ACHD anxiety and depressive symptoms handicap patients to receive medical care. Postponement of medical appointments additionally relates to female sex and documented arrhythmias. The latter indicates that patients at high risk of adverse cardiac outcome avoid routine medical care. Our data may lead policy makers to develop strategies for the provision of medical services to particular vulnerable patient groups, and to optimize management of both future pandemics and daily routine.

## Introduction

Physical and social distancing during the pandemic challenged the whole humankind and resulted in poorer mental health levels (1). Even though, there are chronic diseases, where compared to controls no changes in mental health during the COVID-19 pandemic or even less mental health illnesses were reported (2), notably in particularly vulnerable groups (3, 4) limitations in medical treatment (5, 6), acute care (7–10), screening examinations (11, 12) and higher levels of mental health issues were observed.

In addition to their physical limitations, ACHD patients are burdened by a huge variety of mental diseases, including depression and anxiety disorders as the most common mental illnesses (13–15) that affect about one third to one-half of this patient population (15, 16).

These patients appear to be particularly challenged by the stressor of the global pandemic associated with the fear of contracting COVID-19, resulting in concerns about seeking medical care (17). Postponement of essential medical appointments and interventions might lead to avoidable complications, potentially associated with an adverse outcome.

The aim of this study was to evaluate factors contributing to limited access to medical care and the postponement of medical appointments in ACHD patients during the Covid-19 pandemic.

## Methods

### Study Design

This single center cross-sectional study evaluated the impact of life-style factors, health behavior and mental disorders on medical care during the COVID-19 pandemic. Participants were recruited from the outpatient clinic of the Adult Congenital Heart Disease Centre at Hannover Medical School between August 2020 and March 2021. The local ethics committee approved this study. All patients gave written informed consent. This study is part of the collaboration of the Department of Cardiology and Angiology and the Department of Psychiatry to evaluate the burden of mental illness in cardiovascular diseases (PsyConHeart).

**Figure 1:**
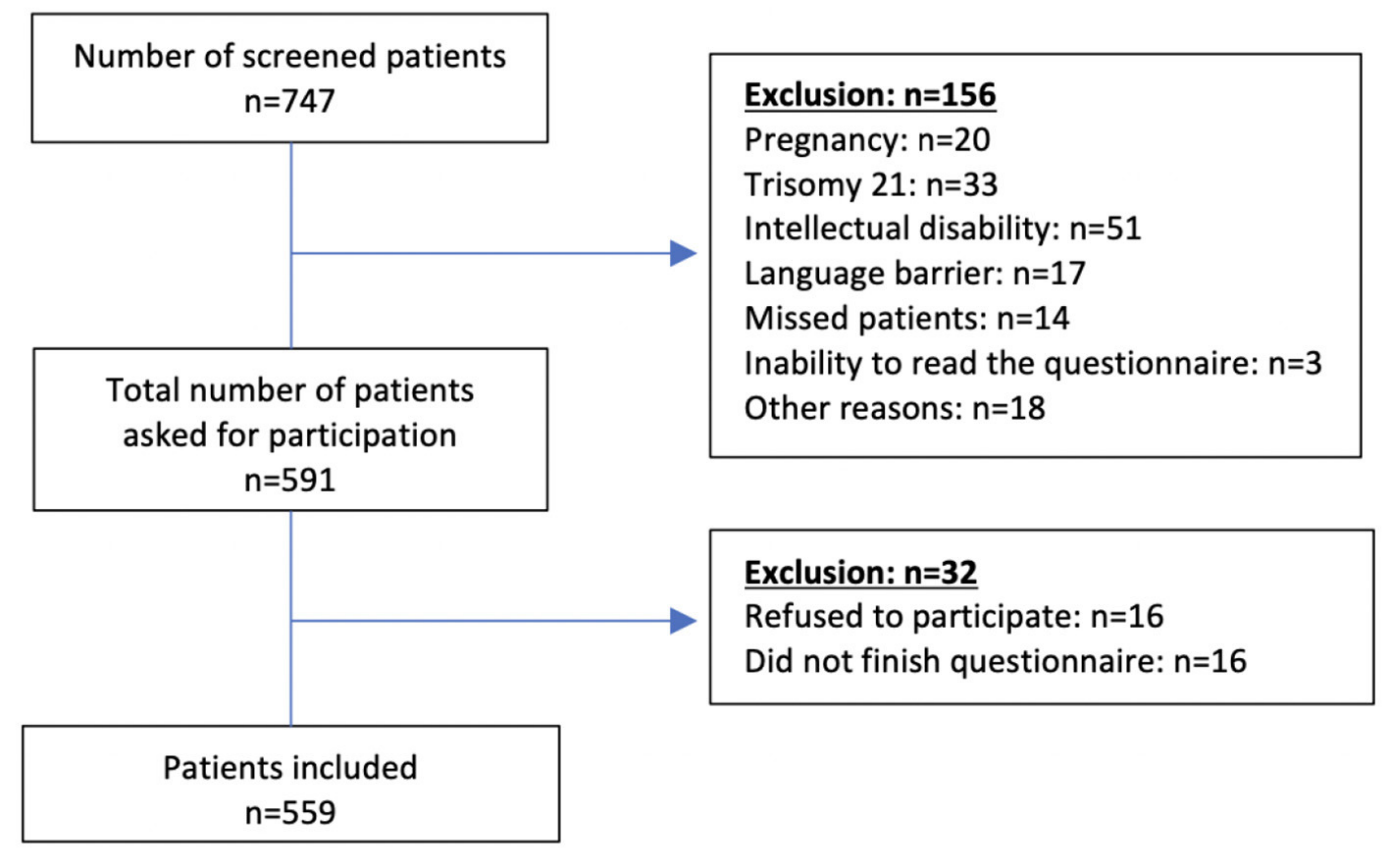
Flowchart of included patients.

Exclusion criteria were pregnancy, intellectual disability, and inability to read and/or to answer the questionnaire. A total number of 591 patients were contacted. Sixteen patients denied participating (2.7%). In addition, 16 patients did not finish the questionnaire, resulting in a dropout rate of 5.4% (Figure 1).

### Data Collection

The remaining 559 patients completed the Hospital Anxiety and Depression Scale (HADS), self- assessment of unhealthy alcohol use (AUDIT), nicotine abuse (Fagerstroem) and internet addiction questionnaire (ISS10). Participants also reported their extent of physical exercise. Further included parameters were self-reported physical limitations using the NYHA-class, the latter differentiated between patients with heart disease without physical limitations in class I, and those with symptoms at rest in class IV. Heart disease complexity was assessed according to the Warnes classification (18). In addition, anthropometric factors, biomarkers, current medication and comorbid physical disorders were included in the analysis. Arrhythmias were classified according to documented episodes of non-sustained supraventricular or ventricular arrhythmias and palpitations, independent from arrhythmia documentation. Patients training at least twice a week were defined as physically active. Details are provided in Table 1.

**Table 1:** Demographic, psychiatric, lifestyle and clinical characteristics of total cohort.

**Parameter**	**Total cohort (*n* = 559)**
Age (years)	37.32 ± 11.98
Male sex (n/%)	296/53
Body Mass Index (kg/m^2^)	*25.81* ± 5.06
Bethesda-classification (I/II/III)^a^	59/201/299
NYHA-Class I/II/ (II/IV) (%)^b^	396/127/34/2
Diabetes (%)	3.2
Hypertension (%)	21.2
Pulmonary arterial hypertension (%)	3.7
Obstructive lung disease (%)	6.8
Oxygen-saturation (%)	97.81 ± 2.49
Hs-CRP (mg/l)^c^	1.94 ± 2.88
Creatinine (μmol/l)	81.85 ± 28.79
GGT (U/l)^d^	36.72 ± 37.82
GPT (U/l)^e^	27.69 ± 16.53
GOT (U/l)^f^	26.3 ± 15.76
GDF15 (ng/l)^g^	541.74 ± 845.27
NT-proBNP (ng/l)^h^	201.23 ± 347.24
HBA1c (%)^i^	5.39 ± 1.3
Hemoglobin	14.5 ± 1.72
QRS (ms)	123.1 ± 85.11
QTc (ms)	438.37 ± 43.65
Intermittent arrhythmias (n/%)	79/14.2
Palpitations (n/%)	50/8.94
ICD (n/%)^a^^0^	20/3.6
Pacemaker (n/%) Limited medical care (n/%)	49/8.7 168/30.1
Perceived limitations in medical treatment (n/%)	109/19.5
Missed medical appointment (n/%)	91/16.3
Insufficient medical treatment (n/%)	63/11.3
Limited drug access (n/%)	59/10.6
AUDIT^k^	2.91 ± 3.04
Smoker (n/%)	65/11.6
ISS 10^l^	14.48 ± 4.22
Non-Adherence (A-14)^m^ n=444	49.57 ± 7.28
Regular Physical activity (n/%)	348/65,2
HADS-A^n^	5.84 ± 3.71
HADS-D^°^	3.77 ± 3.25

a *Classification of heart defect severity*,

b *NYHA: Severity of chronic heart failure according to New York Heart Association*,

c *high sensitivity C-reactive protein*,

d *Gamma-Glutamyltransferase*,

e *Glutamat-Pyruvat-Transaminase*,

f *Glutamat-Oxalacetat-Transaminase*,

g *Growth-Differentiation Factor*,

h *N-terminal prohormone of brain natriuretic peptide*,

i *Hemoglobin A_1c_*,

*^j^Implantable Cardioverter Defibrillator*,

k *alcohol use disorder identification test*,

l *internet addiction scale*,

m *questionnaire for assessment of adherence and individual barriers*,

n *Hospital anxiety scale*,

° *Hospital depression scale*.

We focused on the question whether during the COVID-19 pandemic patients afflicted with congenital heart disease might have concerns about getting in contact with the medical health system and the ability to receive necessary drug treatment. In order to characterize patients with limited access to medical care or postponed medical appointments we dichotomized according to their answers to the following questions:

1. Did you postpone a medical appointment?
2. Did you have limited access to medical treatment and/or delivery of pharmaceuticals?

### Statistical Analysis

Baseline characteristics of the total cohort and the evaluated subgroups are presented in Table 1. Continuous variables are provided as mean and standard deviation, categorical variables as absolute numbers or relative proportions.

We calculated group differences between participants with/ without postponed appointments and limited access to medical care and/or necessary medication.

Unpaired *t*-test, Chi-Square-test and the Man-Whitney–*U*-Test calculated group differences. Binary regression analysis estimated the association between the reported medical care limitations during the COVID-19 pandemic and their explanatory variables.

Logistic regression analysis estimated dependent variables. All single parameters were calculated in univariate analysis, variables with *p* < 0.05 were included in multivariate calculation. The influence of depression and anxiety as measured with the Hospital Anxiety and Depression Scale (HADS) were calculated in different models, due to a close interrelationship of the depression and anxiety subscales (*r* = 0.66). These models also were adjusted for age, sex and BMI to evaluate their potential impact on independent variables.

Statistics was performed with IBM SPSS Statistics® 26.0.

## Results

### Prevalence of Reported Limited Access to Medical Supply

**Figure 2:**
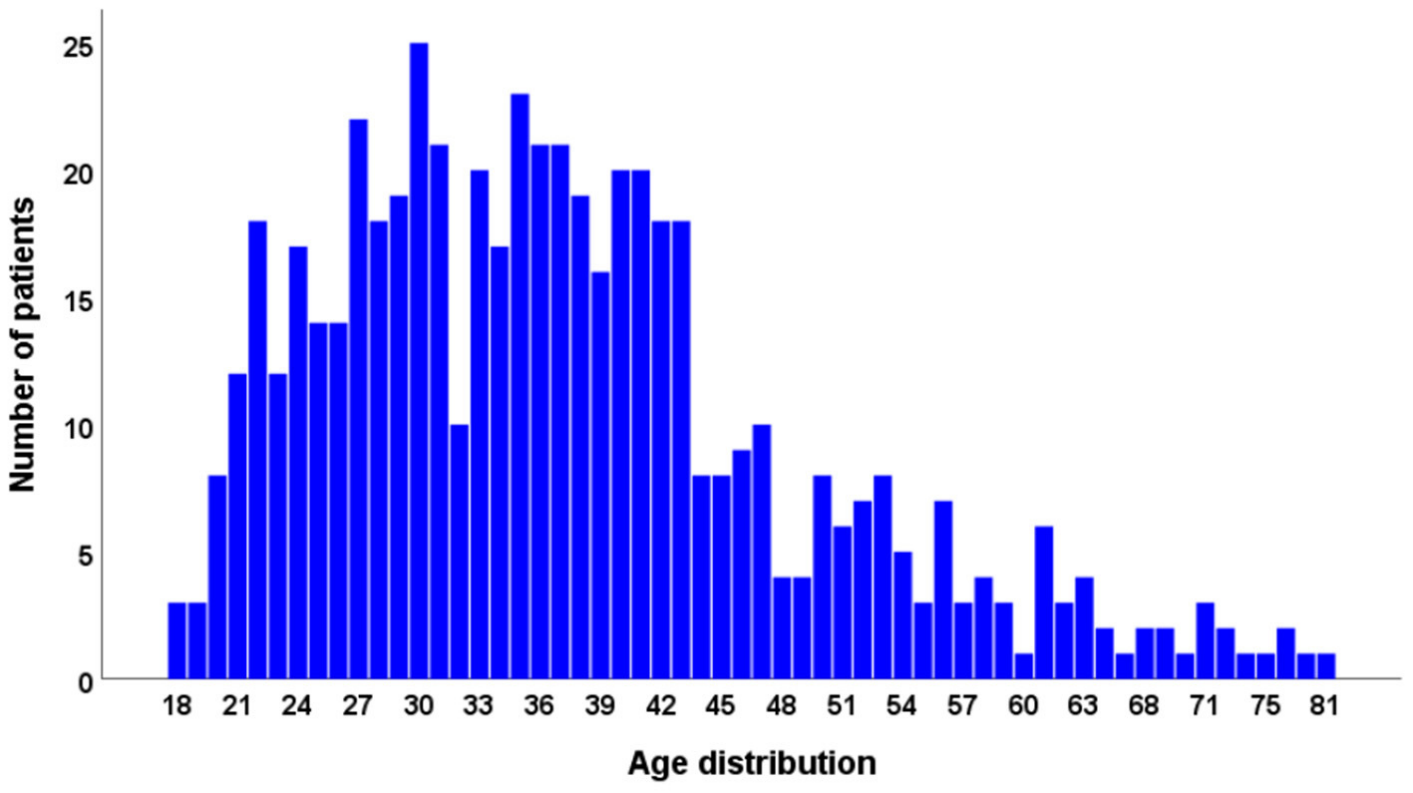
Age distribution.

A total of 559 patients (average age: 37.32 ± 11.98, female sex: 47%) participated in our study (Figure 2). The cohort reporting limited access to either pharmaceuticals (*n* = 59) and/or medical treatment (*n* = 63) consisted of 109 participants (19.5%). Moreover, 91 patients (16.28%) reported postponed appointments, varying between 1 and 10 in number. Of 559 participants, 168 (30.1%) patients reported at least one of these factors.

### Differences in Patients With and Without Limited Access to Medical Treatment/Delivery of Pharmaceuticals

Patients with reported limited access to medical care and/or delivery of pharmaceuticals showed significantly higher self-reported depression (4.41 ± 3.47 vs. 3.62 ± 3.18, *p* = 0.026) and anxiety levels (6.92 ± 3.94 vs. 5.58 ± 3.61, *p* = 0.001) according to the HADS scales. In addittion they reported less physical activity (49.54 vs. 65.33%, *p* = 0.001). Supplementary high sensitivity C-reactive proteine (hs-CRP) was significantly higher in this group (2.46 ± 3.26 vs. 1.82 ± 2.77, *p* = 0.037; Table 2).

**Table 2:** Displays the differences in demographic, psychiatric, lifestyle and laboratory data stratified according to limited or unlimited access to medical treatment.

**Parameter**	**Limited medical treatment** **(*n* = 109)**	**No limited medical treatment** **(*n* = 450)**	***p*-value**
Age (years)	38.59 ± 13.09	37.01 ± 11.69	0.22
Male sex (n/%)	50/45.87	246/54.67	0.11
Body Mass Index (kg/m^2^)	26.52 ± 4.99	25.64 ± 5.07	0.1
Bethesda-classification (I/II/III)^a^	11/38/60	48/163/239	0.72
NYHA-Class I/II/ (II/IV (n)	73/27/9/0	323/100/25/2	0.30
Diabetes (n)	103/6	438/12	0.17
Hypertension (n)	89/20	351/99	0.44
Pulmonary arterial hypertension (n)	106/3	432/18	0.78
Obstructive lung disease (n)	99/10	422/28	0.29
Oxygen-saturation (%)	97.99 ± 1.37	97.76 ± 2.71	0.43
Hs-CRP (mg/l)^c^	2.46 ± 3.26	1.82 ± 2.77	**0.037**
Creatinine (μmol/l)	79.28 ± 16.15	82.47 ± 31.1	0.14
GGT (U/l)^d^	38.61 ± 37.71	36.26 ± 37.87	0.56
GGT (U/l)^d^	28.7 ± 15.91	27.44 ± 16.68	0.46
GPT (U/l)^e^	26.5 ± 8.88	26.25 ± 17.03	0.83
GOT (U/l)^f^	613.31 ± 596.92	524.13 ± 895.56	0.22
NT-proBNP (ng/l)^h^	190.62 ± 312.76	203.86 ± 355.53	0.7
HBA1c (%)^i^	5.46 ± 0.65	5.37 ± 1.41	0.36
Hemoglobin	14.4 ± 1.8	14.53 ± 1.7	0.48
QRS (ms)	120.22 ± 32.75	123.73 ± 93.55	0.52
QTc (ms)	436.18 ± 50.77	438.91 ± 41.77	0.61
Intermittent arrhythmias (n/%)	15/13.76	64/14.22	1
Palpitations (n/%)	15/13.76	35/77.78	0.061
ICD (n/%)^j^	4/3.67	16/3.56	1
Pacemaker (n/%)	12/11.01	37/8.22	0.35
AUDIT^k^	2.39 ± 2.27	3.04 ± 3.19	0.47
Smoker (n/%)	16/14.68	49/10.89	0.32
ISS 10^l^	15.07 ± 4.21	14.34 ± 4.22	0.11
Adherence (A-14)^m^	48.46 ± 7.53	49.84 ± 7.20	0.12
Physical activity (n/%)	54/49.54	294/65.33	**0.001**
HADS-A^n^	6.92 ± 3.94	5.58 ± 3.61	**0.001**
HADS-D^°^	4.41 ± 3.47	3.62 ± 3.18	**0.026**

a *Classification of heart defect severity*,

*^b^NYHA: Severity of chronic heart failure according to New York Heart Association*,

c *high sensitivity C-reactive protein*,

d *Gamma-Glutamyltransferase*,

e *Glutamat-Pyruvat-Transaminase*,

f *Glutamat-Oxalacetat-Transaminase*,

*^g^Growth-Differentiation Factor*,

h *N-terminal prohormone of brain natriuretic peptide*,

i *Hemoglobin A_1c_*,

j *Implantable Cardioverter-Defibrillator*,

k *alcohol use disorder identification test*,

l *internet addiction scale*,

m *questionnaire for assessment of adherence and individual barriers*,

n *Hospital anxiety scale*,

° *Hospital depression scale. Bold numbers represent significant values*.

### Differences in Patients With/Without Postponed Medical Appointments

Female sex turned out to be the main influencing factor of postponed medical appointments (68.13 vs. 43.04%, *p* ≤ 0.0001), significabtly higher depression (4.47 vs. 3.61, *p* = 0.02) and anxiety scores (6.73 vs. 5.65, *p* = 0.01), less physical activity (51.69 vs. 67.79%, *p* = 0.005) and less reported alcohol consumption (2.27 vs. 3.04, *p* = 0.01). However, in all subgroups the reported amount of drinking alcohol reflected a low-risk consumption according to World Health Organization (WHO) guidelines (<7).

Furthermore, we exhibited higher NYHA class (*p* = 0.003), more documented intermittend arrhythmias (24.18 vs. 12.23%, *p* = 0.005) and reported palpitations (15.38 vs. 7.71%, *p* = 0.026). In addition, a lower creatinine level (75.72 vs. 82.92, *p* = 0.03) accompanied postponed appointments, which can be explained by the physiologically lower creatinine observed in female (Table 3).

**Table 3:** Clinical characteristics, demographic, psychiatric, lifestyle and laboratory data of patients' cohort, either keeping or postponing at least one medical appointment.

**Parameter**	**Missed medical appointments** **(*n* = 91)**	**No missed medical appointments** **(*n* = 468)**	***p*-value**
Age (years)	39.71 ± 11.80	36.85 ± 11.98	0.036
Male sex (n/%)	29/31.87	266/56.96	**<0.0001**
Body Mass Index (kg/m^2^)	25.54 ± 4.47	25.85 ± 5.17	0.59
Bethesda-classification (I/II/III)^a^	10/28/53	49/173/245	0.4
NYHA-Class I/II/ (II/IV) (%)^b^	52/32/6/1	343/95/28/1	**0.003**
Diabetes (n)	88/3	452/15	1.0
Hypertension (n)	73/8	367/101	0.78
Pulmonary arterial hypertension (n)	85/6	452/15	0.13
Obstructive lung disease (n)	83/8	437/30	0.37
Oxygen-saturation (%)	97.73 ± 2.94	97.83 ± 2.41	0.79
Hs-CRP (mg/l)^c^	1.88 ± 2.58	1.96 ± 2.94	0.79
Creatinine (μmol/l)	75.72 ± 14.29	82.92 ± 30.64	**0.03**
GGT (U/l)^d^	39.63 ± 43.13	36.17 ± 36.78	0.43
GPT (U/l)^e^	26.08 ± 14.17	27.94 ± 16.92	0.33
GOT (U/l)^f^	24.55 ± 6.37	26.59 ± 16.95	0.26
GDF15 (U/l)^g^	565.87 ± 491.8	537.16 ± 898.33	0.77
NT-proBNP (ng/l)^h^	257.04 ± 452.17	190.87 ± 322.83	0.1
HBA1c (%)^i^	5.44 ± 0.55	5.38 ± 1.40	0.44
Hemoglobin	14.46 ± 1.75	14.51 ± 1.71	0.84
QRS (ms)	116.22 ± 30.40	124.44 ± 92.14	0.4
QTc (ms)	445.22 ± 47.85	437.08 ± 42.74	0.13
Intermittent arrhythmias (n/%)	22/24.18	57/12.23	**0.005**
Palpitations (n/%)	14/15.38	36/7.71	**0.026**
ICD (n/%)^j^	6/6.59	14/3	0.12
Pacemaker (n/%)	7/7.69	41/8.78	0.84
AUDIT^k^	2.27 ± 2.55	3.04 ± 3.12	**0.01**
Smoker (n/%)	15/16.48	50/20.73	0.15
ISS 10^l^	14.84 ± 4.43	14.4 ± 4.18	0.36
Adherence (A-14)^m^	47.83 ± 7.54	49.94 ± 7.18	**0.021**
Physical activity (n/%)	46/51.69	301/67.79	**0.005**
HADS-A^n^	6.73 ± 3.66	5.65 ± 3.69	**0.01**
HADS-D^°^	4.47 ± 3.6	3.61 ± 3.15	**0.02**

a *Classification of heart defect severity*,

b *NYHA: Severity of chronic heart failure according to New York Heart Association*,

c *high sensitivity C-reactive protein*,

d *Gamma-Glutamyltransferase*,

e *Glutamat-Pyruvat-Transaminase*,

f *Glutamat-Oxalacetat-Transaminase*,

g *Growth-Differentiation Factor*,

h *N-terminal prohormone of brain natriuretic peptide*,

i *Hemoglobin A_1c_*,

j *Implantable Cardioverter-Defibrillator*,

k *alcohol use disorder identification test*,

l *internet addiction scale*,

m *questionnaire for assessment of adherence and individual barriers*,

n *Hospital anxiety scale*,

° *Hospital depression scale. Bold numbers represent significant values*.

### Univariate and Multivariate Analysis Predicting Limited Access to Medical Supply

The univariate analysis for limited acces to medical treatment/delivery of pharmaceuticals identified higher HADS-D (HR: 1.073, 95%-CI: 1.008–1.142, *p* = 0.027) and HADS-A levels (HR: 1.096, 95%-CI: 1.037-1-159; *p* = 0.001) as well as increased hs-CRP (HR: 1.069, 95%-CI: 1.002–1.139; *p* = 0.042) as risk factors. Drinking more alcohol (HR: 0.917, 95%-CI: 0.842–0.999; *p* = 0.048) and doing more sports (HR: 0.486, 95%-CI: 0.315–0.75; *p* = 0.001) came up as protective factors, minimizing the risk of a percieved limited access to medical treatment.

In the univariate analysis, risk factors for postponed medical appointments were higher HADS-D (HR: 1.078, 95%-CI: 1.01–1.151; *p* = 0.023) and HADS-A levels (HR: 1.077, 95%-CI: 1.015–1.143; *p* = 0.014), female sex (HR: 2.829, 95%-CI: 1.755–4.561; *p* ≤ 0.0001), higher NYHA class (HR: 1.555, 95%-CI: 1.119–2.163; *p* = 0.009), intermittend arrhythmias (HR: 2.288, 95%-CI: 1.315–3.982; *p* = 0.0039) and palpitations (HR: 2.177, 95%-CI: 1.22–4.225; *p* = 0.0229). In contrast, more physical activity (HR: 0.508, 95%-CI: 0.321–0.806, *p* = 0.004), more alcohol consumption (HR: 0.896, 95%-CI: 0.812–0.988, *p* = 0.028) and higher creatinine levels (HR: 0.976, 95%-CI: 0.961–0.992, *p* = 0.003) acted as protective factors.

Multivariate analysis identified independent predictors of the evaluated subgroups. Due to the close interrelationship of HADS-A and HADS-D (*r* = 0.66) we calculated different models, either including or excluding HADS-D or HADS-A together with all other significant parameters as derived from the univariate analysis.

In unadjusted analysis single predictors for postponed medical apppoinments were female sex (*p* < 0.0001; 0.001), more documented intermittant arrythmias (*p* = 0.007; 0.01) and higher HADS-A (*p* = 0.05) or HADS-D levels (*p* = 0.013). In particular higher levels of physical activity (*p* = 0.02–0.46) acted as a protective factor.

The age and BMI adjusted analysis did not show any differences, regardless of whether the calculation was carried out with HADS-D or HADS-A as a variable. In contrast, including HADS-A in the calculation, adjustment for age and sex identified documented arrhythmias (*p* = 0.005), HADS-A score (*p* = 0.30), and physical activity (*p* = 0.012) as independent variables. Palpitations (*p* = 0.052) and creatinine levels (*p* = 0.051) missed significance level.

The age and sex adjusted analysis of the multivariate model including HADS-D and all other estimated significant univariate parameters computed HADS-D (*p* = 0.033), documented arrhythmias (*p* = 0.012), creatinine (*p* = 0.008) and physical activity (*p* = 0.012) as independent predictors of postponed medical appointments. The observed differences in creatinine levels can be interpreted as a physiological finding, as men have higher creatinine levels.

The analysis regarding limited access to medical supply only partially retrieved similar results. Multivariate analysis of raw data did not identify depression and sex as single predictors, whereas higher anxiety levels (*p* = 0.004) and slightly elevated hs-CRP levels (*p* = 0.03–0.05) remained in the analysis. Physical activity once again came up as a protective factor (*p* = 0.019–0.02). In contrast, adjustment for age and sex estimated HADS-D (*p* = 0.028), HADS-A (*p* = 0.014) and physical activity (*p* = 0.012) as independent predictors. Alcohol consumption remained as an independent predictor in the sex-adjusted model including HADS-D. Adjustment for BMI led to the exclusion of hs-CRP as a significant variable.

Importantly, adherence to treatment was only slightly different between the groups and did not emerge as a predictive factor in uni- or multivariate analysis (Table 4).

**Table 4:** Factors that impact access to medical supply and adherence to medical appointments during the COVID-19 pandemic.

**Binary logistic regression analysis**	**Limited access to medical supply (*****n*** **=** **109)**		**Postponed medical appointments (*****n*** **=** **91)**	
**Univariate Analysis**	**HR^**f**^ (95%CI^**g**^)**	***p*-value**	**HR^**f**^ (95%CI^**g**^)**	***p*-value**
HADS-D^a^	1.073 (1.008–1.142)	**0.027**	1–078 (1.0101–1.151)	**0.023**
HADS-A^b^	1.096 (1.037–1.159)	**0.001**	1.077 (1.015–1.143)	**0.014**
Physical activity	0.486 (0.315–0.750)	**0.001**	0.508 (0.321–0.806)	**0.004**
Hs-CRP^c^	1.069 (1.002–1.139)	**0.042**	0.989 (0.911–1.073)	0.768
Creatinine	0.994 (0.982–1.005)	0.287	0.976 (0.961–0.992)	**0.003**
AUDIT Score^d^	0.917 (0.842–0.999)	**0.048**	0.896 (0.812–0.988)	**0.028**
Sex	0.703 (0.462–1.070)	0.100	2.829 (1.755–4.561)	**<0.0001**
NYHA-Class^e^	0.850 (0.854–1.643)	0.311	1.555 (1.119–2.163)	**0.009**
Documented intermittent arrhythmias	1.042 (0.568–1.909)	0.895	2.288 (1.315–3.982)	**0.003**
Reported palpitations	0.529 (0.277–1.007)	**0.053**	2.177 (1.1221–4.225)	**0.022**
**Multivariate Analysis: HADS-D** ^**a**^				
HADS-D^a^	1.061 (0.994–1.133)	0.074	1.094 (1.019–1.175)	**0.013**
Physical Activity	0.581 (0.367–0.919)	**0.020**	0.598 (0.361–0.990)	**0.046**
AUDIT Score^d^	0.910 (0.830–0.997)	**0.043**		
Hs-CRP^c^	1.070 (1.000–1.144)	**0.050**		
Documented arrhythmias			2.237 (1.217–4.605)	**0.010**
Sex			2.761 (1.656–4.605)	**<0.0001**
**Multivariate Analysis: HADS-A** ^**b**^				
HADS-A^b^	1.089 (1.028–1.154)	**0.004**	1.068 (1.000–1.140)	**0.050**
Physical Activity	0.580 (0.367–0.916)	**0.019**	0.548 (0.330–0.911)	**0.020**
CRP^c^	1.078 (1.007–1.153)	**0.030**		
Documented arrhythmias			2.375 (1.273–4.433)	**0.007**
Sex			2.444 (1.448–4.125)	**0.001**

a *HADS-D: Depression score of HADS*,

b *HADS-A: anxiety score of HADS*,

c *high sensitivity C-reactive protein*,

d *alcohol use disorder identification test*,

e *New York Heart association*,

*^f^Hazard ratio*,

g *Confidence interval. Bold numbers represent significant values*.

### Sex Differences

There were no sex differences in terms of age, HADS-D, heart disease severity and the two interrelated parameters BMI and hs-CRP (*r* = 0.305). Male sex presented significantly higher creatinine levels (89.3 ± 31.6 vs. 73.4 ± 22.5 μmol/l: *p* < 0.001) and AUDIT scores (3.54 ± 3.3 vs. 2.2 ± 2.5; *p* < 0.001). Also, physical activity showed male preponderance (*p* = 0.023). In comparison women had significantly higher anxiety scores (6.3 ± 3.7 vs. 5.4 ± 3.7; *p* = 0.006) and worse functional capacity, regarding NYHA-class (*p* = 0.045). They postponed more medical appointments (*p* < 0.0001), whereas limited access to medical care did not show significant gender differences. Reported palpitations and documented arrhythmias did not show any sex differences.

## Discussion

### Main Findings

In addition to their physical limitations that afford a consequent and specialized treatment (19), ACHD patients are burdened by a huge variety of mental diseases including depression and anxiety disorders as the most common mental illnesses (13–15). Although presumed that in ACHD depression and anxiety symptoms have an impact on attendance, the data from the present study are the first to confirm this assumption.

In general, women presented with significantly higher anxiety symptoms than men. They postponed more medical appointments, pointing to a higher vulnerability of women during the pandemic. It is worrying that patients with documented arrhythmias, which represents a frequent complication being associated with a higher morbidity and mortality risk, were more likely to postpone their medical appointments (20–22).

### Depression and Anxiety as Comorbiditiess

In the present study in univariate analysis, patients with postponed medical appointments and those with limited access to medical treatment/delivery of pharmaceuticals showed significantly higher depression and anxiety levels. In line with previously published data, we found a close interrelationship between anxiety disorders and depression, attributable to shared common biological pathways and behavioral risk factors (23). Nevertheless, in unadjusted analysis we found a preponderance of anxiety symptoms in participants with reported limited access to medical supply, whereas postponed appointments were particularly associated with depression. One might argue, that the described levels of HADS-D and HADS-A are pretty low. However, evalution of the HADS-D score in ACHD establieshed a cut-off point >5 as the optimum of detetecting major depressive disorder (24). In the ACHD population a similar evaluation of the HADS-A score is still missing. Nervertheless, our data indicate that already low levels of anxiety and depressive symptoms are associated with a health behavior potentially linked to adverse cardiac outcome (25).

### Risk Factors of Postponed Medical Appointments

In univariate analysis, postponed medical appointments were associated with more symptomatic heart disease, such as more severe symptoms of heart failure, palpitations and more documented arrhythmias. Irrespective of all adjusting factors, only documented arrhythmias remained as an independent predictor. Additional drivers were higher symptoms of anxiety and depression and female sex.

The postponement of medical appointments in patients with documented arrhythmias represents a finding of particular concern. Arrhythmias in ACHD reflect a major source of morbidity and mortality (20, 21, 26). There is a body of literature pointing to an unfavorable alliance of heart disease and depression/anxiety disorders. The underlying pathophysiology consists of a common biological pathway constituted via the hypothalamic-pituitary-adrenal axis providing bidirectional heart-brain interaction, which modulates cardiopulmonary reflexes and neuroendocrine signaling (23, 27–29). Stress response in anxiety and depression disorders provoke catecholamine related dysregulation of the autonomic nervous resulting in blunted heart rate response, higher heart rate at rest and increased QT-c variability (23). We recently published that ACDH patients with reduced heart rate variability comorbid with depression have a particularly high risk to develop heart failure/all-cause mortality, arrhythmias and unexpected hospitalization due to cardiac causes (30). In non-congenital heart disease, anxiety disorders have been linked to the onset of arrhythmias (31–33). Although the exact mechanism still needs to be determined, cardiac diseases comorbid with depression or anxiety disorders appear to have a higher risk of adverse outcome. Considering that our observations are derived from patients followed by a specialized congenital heart disease center, it is quite likely that outcome is even worse in patients already lost during follow-up (25, 34).

In our study, postponed medical appointments were independently predicted by female sex. During the COVID-19 pandemic observed sex disparities derived from the general population point to a higher vulnerability of women to develop depressive and anxiety symptoms (35).

### Risk Factors of Limited Access to Medical Care

In contrast to patients with postponed medical appointments, those with limited access to medical supply did not suffer from more serious heart defects or symptomatic heart disease. In unadjusted analysis these patients were characterized by significantly higher anxiety symptoms, physical activity, alcohol consumption and the elevated inflammatory marker hs-CRP. However, after adjustment for sex, age and BMI only HADS-D, HADS-A and physical activity remained as independent predictors, whereas higher alcohol consumption and an increase in hs-CRP were no longer significant. Increased hs-CRP levels have been linked to both, depression and anxiety disorders. However, in line with previous reports, our data support the assumption that BMI itself represents the major factor determining hs-CRP elevation. After adjustment with BMI, significant differences were eliminated (36–40).

Raw data analysis also identified higher alcohol consumption as an independent predictor of good medical supply. Male consumed larger amounts of alcohol. However, the overall reported number of drinks (1.8 ± 3.1 drins/week;1 drink=10 g alcohol) was low. In disease entities such as heart failure, frequent consumption of small amounts of alcohol is associated with better outcome, potentially suggesting better social relationships and physical wellbeing (41).

### Protective Effect of Frequent Physical Activity

Our data confirm the assumption that physical activity acts as a protective factor, preventing chronically ill patients from developing threatening fears, such as limited medical care during COVID-19 pandemic or from postponing medical appointments. Previous studies showed a correlation between higher anxiety and depression levels and a lack of physical activity (35), suggesting that increased physical activity might lower the psychological burden in ACHD patients, resulting in less canceled appointments and a higher satisfaction with the level of medical treatment. Moreover, it was described that doing sports according to patients individual physical capacity has a direct positive effect on their health by reducing inflammatory markers and BMI (42). Therefore, it would be desirable to extend sports interventions for ACHD patients (43). However, this inevitably requires an individual assessment of physical capacity as derived from cardiorespiratory exercise testing, which with respect to the underlying cardiac defect determines the extent of exercise training.

### Adherence

As a common feature, adherence to treatment was only slightly different in the evaluated groups, suggesting a general good adherence in ACHD. However, German health insurance-based data approve that at present only half of all German ACHD patients have regular cardiac care (34). Considering that all participants were recruited from a specialized adult congenital heart disease center, our results seem to reflect a selection bias toward better adherence. Previous reports outline that non-attendant patients appear to be linked to non-adherence to medication, which increases the risk of an unfavorable cardiac outcome (25, 44).

In particular, ACHD patients with their congenital heart disease associated long-term sequela and their great burden of mental illness, require essential medical care (15, 19).

It is remarkable, that even in this positive selection of attendant patients during the COVID-19 pandemic depressive and anxiety symptoms led to the postponement of medical appointments and a feeling of insufficient medical care with respect to limited access to medical treatment/delivery of pharmaceuticals. As outlined, ACHD patients with insufficient medical routine examinations and postponed medical appointments tend to have a higher morbidity and mortality (25). Therefore, as proposed in other chronic diseases, identification and treatment of mental health comorbidities, recommendation of remote healthcare support and the encouragement to keep the follow-ups on a regular basis may help to avoid adverse health consequences (45).

### Influence of COVID-19 Pandemic on Lifestyle and Mental Health

The COVID-19 pandemic has put severe stress on particularly vulnerable individuals, such as patients with mental or with physical diseases such as ACHD. Sanctions such as lockdown, no-contact provision, and quarantine may have further increased stress burden in these populations (46). Acute and chronic stress can affect the cardiovascular system and mental well-being of individuals, thereby enhancing the risk for the development or further progression of CVD. This may particularly apply to patients comorbid with physical and mental illness (47). Further, social isolation has dramatically affected lifestyles such as reduced physical activity, sedentary lifestyle, altered dietary habits, and decreased engagement in meaningful activities. Recently, the negative effects of the pandemic and the associated quarantine were reviewed, pointing out that distress caused by social isolation not only worsens mental health, but also negatively affects lifestyle factors (48).

## Conclusion

This is the first study approving that in ACHD anxiety and depressive symptoms directly impact medical care. ACHD patients are burdened by their inborn congenital heart defect related long-term sequela and a high prevalene of mood disorders (15). Depressive and anxiety symptoms are associated with limited access to medical care and the postponement of medical appointments. Female sex and the worrying finding that patients with documented arrhythmias, indicating a higher morbidity and mortality risk, were particularly prone to cancel medical appoinments.

It appears, that advanced symptomatic heart disease comorbid with depression characterizes a particular subgroup of patients at high risk to get lost during follow-up which is potentially asssociated with adverse outcome. Considering the observed inadaquate care of mood disorders in ACHD, regular screning for depressive and anxiety symptoms appears to be advisable (15). In contrast, frequent physical activity emerged as a protective factor.

Our data confirm the need to screen for mental disorders to facilitate psychological treatment (potentially in form of telehealth or digital health advises) to limit anxiety and depression levels, probably leading to less canceled medical appointments and an improved medical treatment (45).

Moreover, we recommend to encourage patients to increase their physical activity, which generates a direct positive effect on their health and additionally works as a protective factor by minimizing anxiety and depressive symptoms (43).

We hypothesize that these interventions might have the potential to improve medical treatment and reduce the number of canceled medical appointments not just during pandemics but also in everyday clinical practice.

## Strengths And Limitations

Although the COVID-19 pandemic offers the chance to exhibit problems in the medical care of ACHD patients, the lockdowns and restrictions caused a selection bias toward the exclusion of patients, who missed their scheduled appointment either suffering from anxiety disorders and depression or other limitations, hindering them from coming to the outpatient department. A further selection bias is given by the monocentric study design.

Moreover, the question regarding limited access to medical treatment and/or delivery of pharmaceuticals comprehends subjective feelings reflecting different perceptions.

As an advantage, the overall drop-off rate was low. Due to the large number of participants, application of standardized questionnaires and inclusion of a cross-sectional population, typically found in ACHD studies, the data appear to be representative for patients followed in a specialized adult congenital heart disease center.

## Data Availability Statement

The raw data supporting the conclusions of this article will be made available by the authors, without undue reservation.

## Ethics Statement

The studies involving human participants were reviewed and approved by Ethics Committee Hannover Medical School Carl-Neuberg-Straße 1, D-30625 Hannover. The patients/participants provided their written informed consent to participate in this study.

## Author Contributions

KK and MW-B are leading investigators in the PsyConHeart study and have been involved in all aspects of this study. In particular, they were involved in (i) experiment design, (ii) experiment realization, (iv) data collection, (v) data analysis, (vi) data interpretation and (vii) manuscript writing. TH and TK were involved in experiment design, data collection, data analysis, and data interpretation and manuscript writing. SA was involved in experiment design, data collection, data analysis, and data interpretation and manuscript writing. FL was involved in data collection and data interpretation and revising the manuscript. TK, AS-P, and JB were involved in data interpretation and revising the manuscript. All authors have materially participated in the research and/or article preparation and have approved the final article.

## Funding

The study was supported by an unrestricted grant from the MHH plus Förderstiftung.

## Conflict of Interest

The authors declare that the research was conducted in the absence of any commercial or financial relationships that could be construed as a potential conflict of interest.

## Publisher's Note

All claims expressed in this article are solely those of the authors and do not necessarily represent those of their affiliated organizations, or those of the publisher, the editors and the reviewers. Any product that may be evaluated in this article, or claim that may be made by its manufacturer, is not guaranteed or endorsed by the publisher.
